# *De Novo* Assembly and Annotation of the Chinese Chive (*Allium tuberosum* Rottler ex Spr.) Transcriptome Using the Illumina Platform

**DOI:** 10.1371/journal.pone.0133312

**Published:** 2015-07-23

**Authors:** Shu-Mei Zhou, Li-Mei Chen, Shi-Qi Liu, Xiu-Feng Wang, Xiu-Dong Sun

**Affiliations:** 1 State Key Laboratory of Crop Biology, College of Life Science, Shandong Agricultural University, Tai’an, Shandong, People's Republic of China; 2 State Key Laboratory of Crop Biology, Key Laboratory of Biology and Genetic Improvement of Horticultural Crops (Huanghuai Region), College of Horticulture Science and Engineering, Shandong Agricultural University, Tai’an, Shandong, People's Republic of China; CNRS UMR7622 & University Paris 6 Pierre-et-Marie-Curie, FRANCE

## Abstract

Chinese chive (*A*. *tuberosum* Rottler ex Spr.) is one of the most widely cultivated *Allium* species in China. However, minimal transcriptomic and genomic data are available to reveal its evolution and genetic diversity. In this study, de novo transcriptome sequencing was performed to produce large transcript sequences using an Illumina HiSeq 2000 instrument. We produced 51,968,882 high-quality clean reads and assembled them into 150,154 contigs. A total of 60,031 unigenes with an average length of 631 bp were identified. Of these, 36,523 unigenes were homologous to existing database sequences, 35,648 unigenes were annotated in the NCBI non-redundant (Nr) sequence database, and 23,509 unigenes were annotated in the Swiss-Prot database. A total of 26,798 unigenes were assigned to 57 Gene Ontology (GO) terms, and 13,378 unigenes were assigned to Cluster of Orthologous Group categories. Using the Kyoto Encyclopedia of Genes and Genomes (KEGG) pathway database, we mapped 21,361 unigenes onto 128 pathways. Furthermore, 2,125 sequences containing simple sequence repeats (SSRs) were identified. This new dataset provides the most comprehensive resource currently available for gene expression, gene discovery, and future genomic research on Chinese chive. The sequence resources developed in this study can be used to develop molecular markers that will facilitate further genetic research on Chinese chive and related species.

## Introduction

Chinese chive (*A*.*tuberosum* Rottler ex Spr.) is a perennial plant that is widely cultivated worldwide. It is commonly used as a spice in Asian cuisines, especially in China, Japan, and Korea. Chinese chive is rich in carbohydrates, proteins, mineral salts and vitamins. As a member of the *Allium* family, Chinese chive contains high concentrations of organic sulfur compounds, which confer characteristic flavors [1] and human health benefits [2]. Chinese chive has been used as a traditional medicine for the treatment of common colds, headaches, and cardiovascular diseases such as increased reactive oxygen species, high blood pressure, high cholesterol, platelet aggregation, and blood coagulation.

The genomes of many *Allium* species are very large relative to other eukaryotes; in 30 *Allium* species, the genome size ranges from 6860 to 30,870 Mbp per 1C. Chinese chive is a tetraploid (2n = 4x = 32) plant with a nuclear genome of 15G per 1C. Its genome is slightly smaller than the onion genome, 30 times larger than the rice genome and approximately 100 times larger than the *Arabidopsis thaliana* genome. Molecular markers, specific functional genes and other genomic resources in Chinese chive are very limited compared with other vegetable taxa such as the gourd and solanaceous vegetables. Transcriptome sequencing is a cost-effective and frequently used strategy for the genome-wide quantification of absolute transcript levels, the development of molecular markers, and the identification of transcripts [3–5].

In recent years, the emergence of next generation sequencing (NGS) technology has offered a powerful and cost-efficient tool for the generation of transcriptomic datasets in non-model species using various platforms such as the Roche 454, Illumina HiSeq, and Applied Biosystems SOLiD [6, 7]. RNA sequencing has been used for the genome-wide quantification of absolute transcript levels, the identification of novel genes, the delineation of transcript structure (including 5′ and 3′ ends, introns, and exons) and the mining of molecular markers [4, 8, 9]. Several non-model organisms such as the Jerusalem artichoke, Sophora japonica, and Youngia japonica have been studied by transcriptome sequencing [3, 6, 10], which has provided a better understanding of these plants.

In the present study, we used the Illumina HiSeq 2000 platform to develop the Chinese chive transcriptome dataset. Raw reads comprising 4.95 Gbp were assembled de novo into 53,837 unigenes. The assembled unigenes were annotated against public protein databases followed by GO, COG and KEGG classification. Moreover, 2,453 simple sequence repeats (SSRs) were identified. The transcriptome data generated in this study provide an invaluable genomic resource for future research on Chinese chive. Additionally, the SSR markers developed here should facilitate marker-assisted selective breeding, gene mapping and linkage map development in Chinese chive.

## Materials and Methods

### Ethics statement

All of the necessary permits for field studies were obtained. The authority responsible for *A*. *tuberosum* farming, Shandong Agricultural University, provided permission to collect the samples for our research.

### Plant materials and RNA extraction

*A*. *tuberosum* seedlings were grown in the fields of the College of Horticulture Science and Engineering of Shandong Agricultural University in Tai’an, Shandong Province, China, under normal cultivation conditions. Leaves, shoots and roots were collected, and the tissues were then immediately frozen and stored in liquid nitrogen. Total RNAs were extracted using TRIzol Reagent and then treated with DNase I according to the manufacturers’ instructions. The RNA quality was verified using a 2100 Bioanalyzer and gel electrophoresis, and the library was sequenced on an Illumina HiSeq 2000 platform.

### Library construction and Illumina sequencing

Purification of the mRNAs was performed using the OligoTex mRNA mini kit mRNAs were chemically fragmented into short pieces using RNA Fragmentation Reagent, and cDNAs were synthesized using the mRNA fragments as templates. The cDNA fragments were purified and dissolved in EB buffer for end repair and single-nucleotide A (adenine) addition. The short fragments were then ligated with sequencing adaptors, and the products were purified and enriched by PCR to generate the final library. The library was sequenced on an Illumina HiSeq 2000 platform. Before assembly, the raw reads were filtered by removing adaptors and low-quality sequences with unknown nucleotides larger than 5% and low quality reads which the percentage of low quality bases (base quality≤10) is more than 20%. De novo assembly of the clean reads was conducted with the short-read assembly program “Trinity” (Release 2013-02-05) [11]. The parameters for Trinity were as follows: seqType fq, min_contig_length 100, min_glue 3, group_pairs_distance 250, path_reinforcement_distance 85, min_kmer_cov 3, SS_lib_type FR.

Trinity combines three independent software modules: Inchworm, Chrysalis, and Butterfly. Inchworm assembles reads into linear transcript contigs in the following steps. Constructs a k-mer dictionary from all sequence reads (in practice, k = 25); Selects the most frequent k-mer in the dictionary to seed a contig assembly (excluding both low-complexity and singleton k-mers); extends the seed in each direction by finding the highest occurring k-mer with a k − 1 overlap until it cannot be extended further, then reports the linear contig; repeats the above two steps, starting with the next most abundant k-mer, until the entire k-mer dictionary has been exhausted. Next, Chrysalis clusters the Inchworm Contigs into clusters and constructs complete de Bruijn graphs for each cluster. Each cluster represents the full transcriptonal complexity for a given gene (or sets of genes that share sequences in common). Chrysalis then partitions the full read set among these disjoint graphs. Finally, Butterfly processes the individual graphs in parallel, tracing the paths that reads and pairs of reads take within the graph, ultimately reporting full-length transcripts for alternatively spliced isoforms, and teasing apart transcripts that corresponds to paralogous genes. The result sequences of trinity is called Unigenes.

To compare unigenes from A. tuberosum and other Alliums, sequence datasets were downloaded from NCBI Transcriptome Shotgun Assembly database (http://www.ncbi.nlm.nih.gov/genbank/tsa/) with the accession numbers (Garlic: TSA JV230866-JV310008; Bunching onion: TSA FX553726-FX608587, FX657476-FX657516; Onion: GBRQ01000000)[12, 13, 14].

### Sequence annotation

The assembled unigenes were used for BLASTn searches and annotation against the NCBI non-redundant nucleotide sequence (Nt) database with an E-value cut-off of 10–5. BLASTx alignment (E-value <1e-5) was performed between the unigenes and the protein databases, including Nr (last updated in March of 2013), the Swiss-Prot protein (Release 2013_03), KEGG (Release 63.0), and COG database (last updated in September of 2009). With Nr annotation, we used the Blast2GO program [15] to predict GO terms related to molecular functions, cellular components, and biological processes. After obtaining GO annotations for every unigene, we used the WEGO software [16] to conduct GO functional classification for all unigenes and to understand the distribution of gene functions throughout the species at the macro level.

### SSR detection and validation

SSRs were detected using the MISA program (http://pgrc.ipk-gatersleben.de/misa/). The minimum repeat number was set to six for di-nucleotides, to five for tri- and tetra-nucleotides and to four for penta- and hexa-nucleotides. Primer3 software was used to design the primer pairs. The major parameters for primer pair design were set as follows: no SSRs were present in the primer; primers aligned to unigene sequences with the 5' site were allowed 3 mismatches; primers aligned to unigene sequences with the 3' site were allowed 1 mismatch; primers that aligned to more than one unigene were removed; SSRs were validated using SSR-finder (http://www.fresnostate.edu/ssrfinder/); and both-hit primers were selected.

## Results and Discussion

### Illumina sequencing and *de novo* assembly

To generate a global overview of the A. tuberosum transcriptome, sequence analysis and assembly were performed using the Illumina HiSeq 2000 sequencing platform. After stringent quality assessment and data filtering, 51,968,882 clean paired-end sequence reads (NCBI SRA accession no. SRR1020564) with total of 4,677,199,380 nucleotides (nt) were produced with an average length of 90 bps for each short read (Table 1). The average GC content of the clean reads was 43.86%. Q20, the proportion of nucleotides with quality value larger than 20 in reads, was 97.81%.

**Table 1:** Summary of the Chinese chive transcriptome sequencing using the Illumina HiSeq.

	Chinese chive
**Total Raw Reads**	57,019,902
**Total Clean Reads**	51,968,882
**Total Clean Nucleotides**	4,677,199,380
**Q20 percentage**	97.81%
**N percentage**	0.00%
**GC percentage**	43.86%

**Fig 1:**
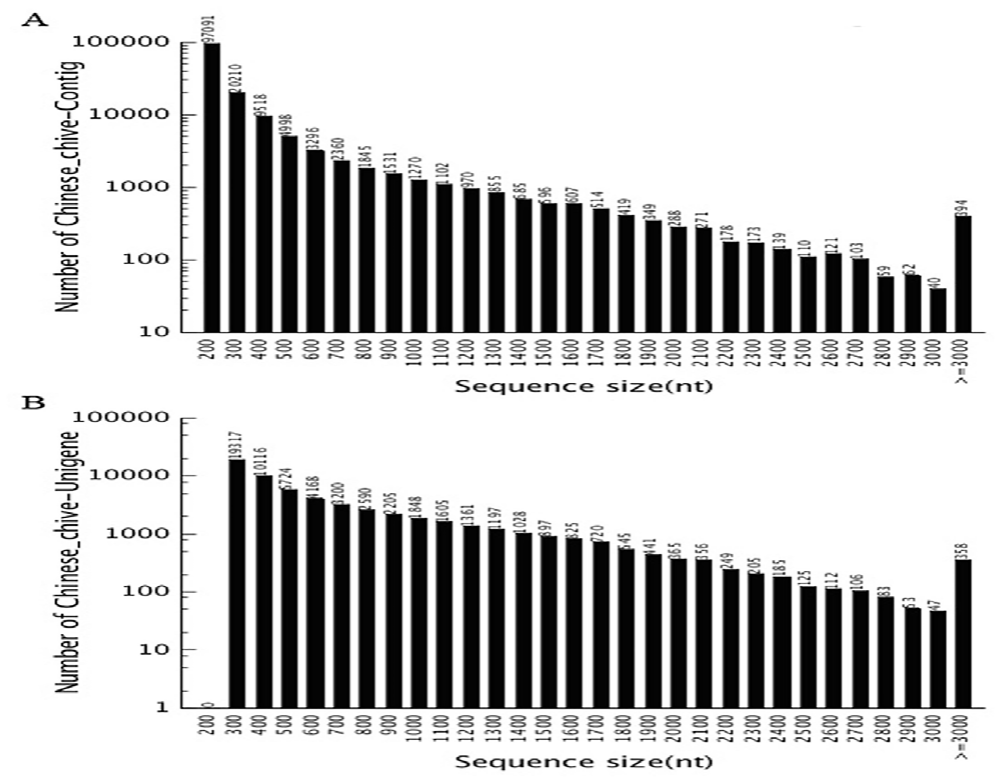
Overview of the transcriptome assembly for *A*. *tuberosum* Rottler ex Spr. (A) Size distribution of contigs; (B) size distribution of unigenes.

Using the Trinity program, the obtained short-read sequences were assembled into 150,154 contigs with an average length of 289 bp and an N50 length of 444 bp. A total of 18,528 contigs, which accounted for 12.21% of the contigs, were longer than 500 bp (Fig 1). The contigs were further clustered and assembled, resulting in 60,031 unigenes, among which 10,863 genes (18.09%) were longer than 1 kb. The average length of these unigenes was 631 bp, and the N50 length was 900 bp (Table 2). The length distributions of the contigs and unigenes are shown in Fig 1. The results suggest that the sequencing data of the Chinese chive transcriptome were effectively assembled. These results also indicate that the throughput and sequencing quality was high enough for further analysis. Because of the relatively large genome sizes of *Allium* species, full-genome sequencing has not been conducted in these species. Transcriptome sequencing has offered a new avenue for generating abundant sequence information from any organism [17, 18]. Transcriptome sequencing has been recently applied to several *Allium* species. A total of 127,933 garlic unigenes with an average length of 363 bp were generated by de novo assembly [12]. A set of 42,881 unigenes with an average length of 787.30 bp were obtained from Welsh onion [19]. A total of 165,179 unigenes with an average length of 1,228.9 bp were generated from onion [20]. Kamenetsky et al [21] generated 239,116 contigs with an average length of 715 bp from garlic. Our reads roughly in the middle as compared to the average lengths of unigenes or contigs obtained from these *Allium* species. The data obtained from RNA-Seq analyses will provide an important basis for future gene cloning and transgenic engineering studies.

**Table 2:** Statistical summary of the de novo transcriptome assembly for *A*. *tuberosum* Rottler ex Spr.

	Total number	Total length	Mean length	N50
**Contig**	150,154	43,413,146	289	444
**Unigene**	60,031	37,900,663	631	900

### Functional annotation and classification

The unigenes were annotated by aligning them with several protein databases, including the Nr database, Nt database, Swiss-Prot, KEGG, COG, and Gene Ontology. In total, 36,523 unigenes were annotated to the six databases (S1 Table). The Annotation Rate of Chinese Chive unigenes was 60.84%, which was higher than Garlic (48.31%) and Onion (58.85%) (Table 3). A total of 23,508 unigenes did not significantly match to any known protein in the public databases. Similar search outcomes have been observed in other studies [3, 6]. These unigenes may be novel transcribed sequences in the *Allium* species. Some unigenes may have also been too short to allow for statistically meaningful matches. As shown in Table 3 35,648 unigene matches were found in the Nr database, 22,798 unigenes were successfully annotated in the Nt database, and 23,509 unigenes were similar to proteins in the Swiss-Prot database.

**Table 3:** Summary of the functional annotation of assembled unigenes.

Category	Chinese Chive	Bunching Onion	Garlic	Onion
**NR Annotation**	35,648	30,751	31,879	90,178
**NT Annotation**	22,798	27,371	20,791	64,670
**Swiss-Prot Annotation**	23,509	19,297	19,425	58,650
**KEGG Annotation**	21,361	17,721	18,084	55,849
**COG Annotation**	13,378	11,680	9,746	34,838
**GO Annotation**	26,798	21,019	23,738	62,250
**ALL Annotations**	36,523	36,351	35,778	97,205
**Total Genes**	60031	54,903	79,143	165,179
**Annotation Rate**	60.84%	66.21%	45.21%	58.85%

**Fig 2:**
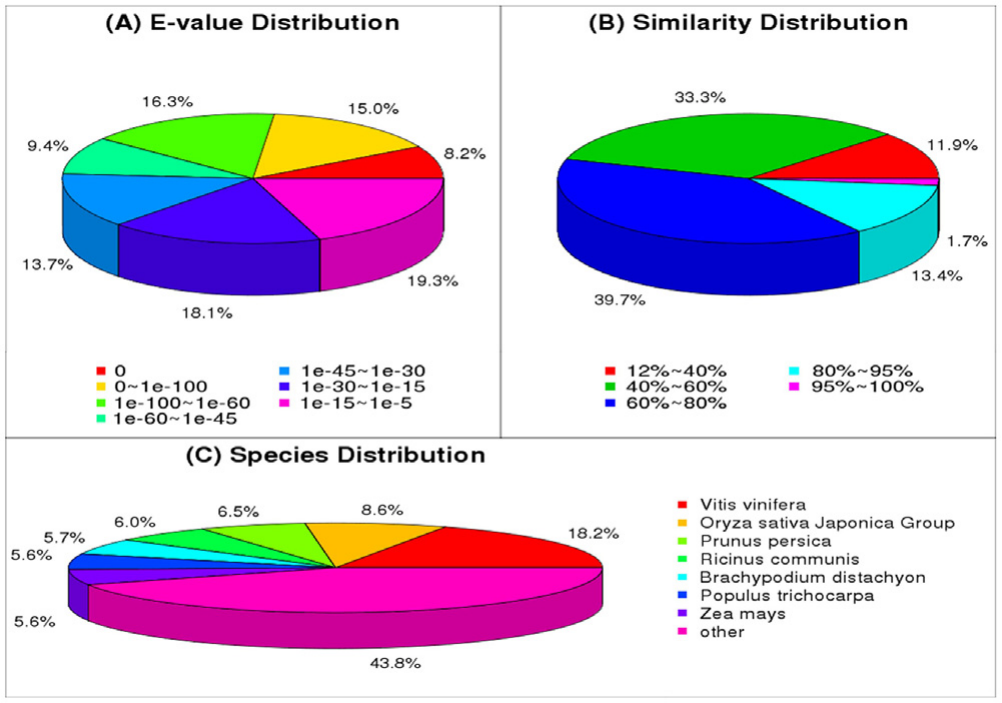
Unigene homology searches against the NR database. (A) The E-value distribution of BLAST hits for the assembled unigenes in the NR database. (B) The similarity distribution of BLAST hits against the NR database for each unigene. (C) Species distribution of the top BLASTx hits against the NR database for each unigene.

The E-value distribution of the top matches showed that 80.72% of the Nr-mapped sequences had values in the range of 0−1.0 x 10^−30^, and 62.58% of the unigenes had a high E-value score (E-value < 10^−45^) (Fig 2A). These results reflect the validity and reliability of our *de novo* assembly, suggesting that the sequences have a good assembling quality. The distribution of sequence similarities showed that 88.10% of the Nr-annotated sequences had similarities greater than 40%, and 15.13% of the sequences shared more than 80% similarity with known sequences (Fig 2B). Additionally, the unigenes were compared to sequences of other plant species; 6,502 (18.2%) unigenes were best matched to sequences from *Vitis vinifera*, whereas 3,063 (8.6%), 2,317 (6.5%), 2,145 (6.0%), 2,016 (5.7%), 2,004 (5.6%), and 1,990 (5.6%) were matched to sequences from Oryza sativa Japonica Group, *Prunus persica*, *Ricinus communis*, *Brachypodium distachyon*, *Populus trichocarpa*, and *Zea mays*, respectively (Fig 2C).

### Gene Ontology (GO) classification

**Fig 3:**
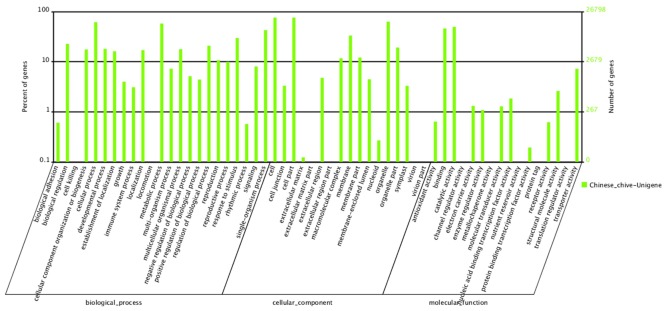
GO classification of assembled sequences. A total of 13,897 unigenes were grouped into three main GO categories: ‘Biological Processes’, ‘Cellular Component’, and ‘Molecular Function’.

To classify the predicted functions of the assembled unigenes, the Blast2GO program [15] was utilized. Based on sequence homology, GO classification revealed that 26,798 (44.64%) sequences could be categorized into 56 functional groups (Fig 3). In the Biological Processes category, cellular process (16,492, 61.54%), metabolic process (15,508, 57.87%), single-organism process (11,450, 42.74%), response to stimulus (7,968, 29.73%) and biological regulation (6,088, 22.72%) were prominently represented. Within the Cellular Component category, cell (20,370, 76.01%), organelle (16,893, 63.04%) and membrane (8,920, 33.29%) were the most highly represented groups. Under the Molecular Function category, catalytic activity (13,309, 49.66%), binding (12,362, 46.13%) and transporter activity (1,941, 7.24%) were prominently represented. These results were slightly different from those obtained for *Youngia japonica* and *Auricularia polytricha* [8, 10]. These GO annotations provide comprehensive information on the transcript functions of the *A*. *tuberosum*.

### COG classification

**Fig 4:**
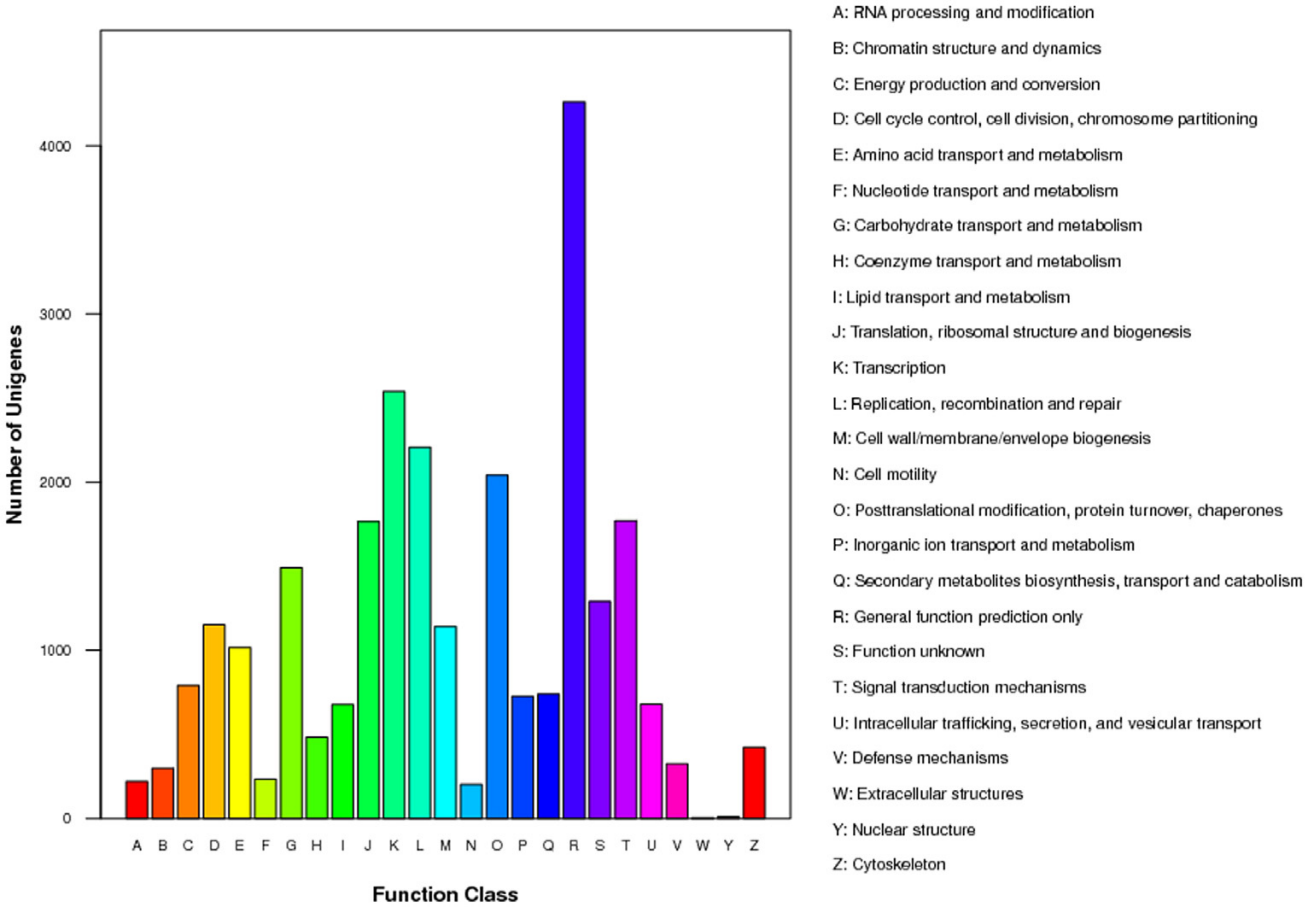
COG functional classification of unigenes. A total of 950 assembled unigenes were annotated and assigned to 24 functional categories.

The COG database is used to phylogenetically classify proteins that are encoded in completely sequenced genomes. Of the 60,031 unigenes, 13,378 (22.29%) were annotated and classified into 25 functional categories (Fig 3). The identity ratio in our study was higher than 3.63% in *Ziziphus jujube* [22], higher than 5.15% in *Lycoris aurea* [23] and less than 24.42% in rubber tree [24]. Among the aligned COG classifications, the category of general function prediction comprised the largest group (4,260, 31.84%), followed by transcription (2,539, 18.98%), replication, recombination and repair (2,208, 16.50%), posttranslational modification, protein turnover and chaperones (2,042, 15.26%), signal transduction mechanisms (1,771, 13.24%), translation, ribosomal structure and biogenesis (1,766, 13.20%), and carbohydrate transport and metabolism (1,492, 11.15%). In addition, 1291 unigenes were assigned to the unknown function classification. The two categories comprising nuclear structure and extracellular structures comprised 10 (0.07%) and 4 (0.03%) unigenes, respectively, representing the two smallest COG categories (Fig 4).

### KEGG Classification

The KEGG database contains data from a systematic analysis of inner-cell metabolic pathways and functions of gene products. Pathway-based analysis is helpful for understanding the biological functions and interactions of genes [25]. A total of 21,361 annotated unigenes were found to have significant matches in the KEGG database and were assigned to 128 known biological pathways (S2 Table). The pathways with the most annotated genes were metabolic pathways (5002 unigenes, 23.42%, ko01100), followed by biosynthesis of secondary metabolites (2342 members, 10.96%, ko01110), plant-pathogen interaction (1041 members, 4.87%, ko04626), plant hormone signal transduction (1013 members, 4.74%, ko04075), RNA transport (883 members, 4.13%, ko03013), spliceosome (816 members, 3.82%, ko03040), endocytosis (808 members, 3.78%, ko04144), glycerophospholipid metabolism (744 members, 3.48%, ko00564), and starch and sucrose metabolism (704 members, 3.3%, ko00500). Similar results were obtained in other studies [14, 22]. The predicted metabolic pathways are useful for future research into gene functions.

### SSR discovery

Using MISA software, the assembled sequences were scanned to explore SSR profiles. In total, 2,125 sequences containing 2,279 potential SSRs were identified from the 60,031 assembled sequences. The percentage (3.8%) of mined SSRs in this study was similar to those in the reports for other Lilium species and cultivars [26, 27]. A total of 142 sequences contained more than one SSR, and 79 SSRs were present in compound formation. On average, the SSR frequency in the Chinese chive transcriptome was 3.80%, and one SSR could be found every 16.63 kb in the transcriptome. The tri-nucleotide SSRs (1,100, 48.27%) were the most abundant, followed by mono-nucleotide (611, 26.81%) and di-nucleotide repeat motifs (477, 20.93%), whereas hexa-nucleotide (55, 2.41%), quad-nucleotide (21, 0.92%), and penta-nucleotide repeats (15, 0.66%) were rare (Table 4). The most abundant motif in the dinucleotide class was AC/GT (273, 13.56%), followed by AG/CT (231, 11.47%), AT/AT (97, 4.82%) and the least represented motif was CG/CG (10, 0.5%) (Table 5). The dominant repeat motifs in the tri-nucleotide class was AAG/CTT (303, or 13.30%), ATC/ATG (174, or 8.64%), AGC/CTG (155, or 7.7%) and AGG/CCT (154, or 7.65%), as shown in Table 5. All of the above tri-nucleotide repeats comprised 71.47% of the characterized tri-nucleotides. For Chinese chive, SSR lengths ranged from 12 to 136 nt. The majority of tri-nucleotide repeats lengths ranged from 15 to 30 bp (data not shown). A total of 1,937 primer pairs were specifically designed from 2,125 sequences (S3 Table), which provide a good resource for molecular marker-assisted breeding.

**Table 4:** Statistics of the SSRs identified in the *A*. *tuberosum* transcriptome.

Item	Numbers
**Total number of sequences examined**	60031
**Total size of examined sequences (bp)**	37900663
**Total number of identified SSRs**	2279
**Number of SSR-containing sequences**	2125
**Number of sequences containing more than 1 SSR**	142
**Number of SSRs present in compound formation**	79
**Number of mono-nucleotide repeats**	477
**Number of di-nucleotide repeats**	611
**Number of tri-nucleotide repeats**	1,100
**Number of quad-nucleotide repeat**	21
**Number of penta-nucleotide repeats**	15
**Number of hexa-nucleotide repeats**	55

**Table 5:** Frequency of dinucleotide and trinucleotide SSRs repeat motifs in *A*. *tuberosum*.

Repeats	5	6	7	8	9	>9	Total	%
**AC/GT**		95	54	40	23	61	273	11.98
**AG/CT**		90	67	40	12	22	231	10.14
**AT/AT**		53	10	9	5	20	97	4.26
**CG/CG**		7	3				10	0.44
**AAC/GTT**	40	14	7	2			63	2.76
**AAG/CTT**	193	71	34	5			303	13.3
**AAT/ATT**	54	17	13	4			88	3.86
**ACC/GGT**	65	19	5	1			90	3.95
**ACG/CGT**	17	7	1				25	1.1
**ACT/AGT**	7	1	2	4			14	0.61
**AGC/CTG**	85	44	20	6			155	6.8
**AGG/CCT**	98	36	18	2			154	6.76
**ATC/ATG**	112	30	27	5			174	7.63
**CCG/CGG**	28	4	1	1			34	1.49

## Supporting Information

S1 Table Unigene annotation by the NCBI Nr and Nt, Swiss-Prot, KEGG, COG and GO databases. (XLS)

S2 Table List of KEGG pathways involving Chinese chive unigenes. (DOC)

S3 Table Designed SSR primers for Chinese chive. (XLS)
