# Supplementary material for: De Novo Assembly and Annotation of the Chinese Chive (Allium tuberosum Rottler ex Spr.) Transcriptome Using the Illumina Platform
Source: PLoS One. 2015 Jul 23;10(7):e0133312. doi: 10.1371/journal.pone.0133312 (PMC4512717; doi:10.1371/journal.pone.0133312)
Supplement: S2 Table — (DOC) [file pone.0133312.s002.doc]

Table S2. List of KEGG pathways in Chinese chive unigenes.

| # | Pathway | All genes with pathway annotation (21361) | Pathway ID |
| --- | --- | --- | --- |
| 1 | Metabolic pathways | 5002 (23.42%) | ko01100 |
| 2 | Biosynthesis of secondary metabolites | 2342 (10.96%) | ko01110 |
| 3 | Plant-pathogen interaction | 1041 (4.87%) | ko04626 |
| 4 | Plant hormone signal transduction | 1013 (4.74%) | ko04075 |
| 5 | RNA transport | 883 (4.13%) | ko03013 |
| 6 | Spliceosome | 816 (3.82%) | ko03040 |
| 7 | Endocytosis | 808 (3.78%) | ko04144 |
| 8 | Glycerophospholipid metabolism | 744 (3.48%) | ko00564 |
| 9 | Starch and sucrose metabolism | 704 (3.3%) | ko00500 |
| 10 | Protein processing in endoplasmic reticulum | 627 (2.94%) | ko04141 |
| 11 | Ether lipid metabolism | 588 (2.75%) | ko00565 |
| 12 | Ribosome | 573 (2.68%) | ko03010 |
| 13 | RNA degradation | 506 (2.37%) | ko03018 |
| 14 | Ribosome biogenesis in eukaryotes | 495 (2.32%) | ko03008 |
| 15 | mRNA surveillance pathway | 456 (2.13%) | ko03015 |
| 16 | Purine metabolism | 446 (2.09%) | ko00230 |
| 17 | Ubiquitin mediated proteolysis | 426 (1.99%) | ko04120 |
| 18 | Pyrimidine metabolism | 391 (1.83%) | ko00240 |
| 19 | Pentose and glucuronate interconversions | 349 (1.63%) | ko00040 |
| 20 | Phenylpropanoid biosynthesis | 340 (1.59%) | ko00940 |
| 21 | Glycolysis / Gluconeogenesis | 312 (1.46%) | ko00010 |
| 22 | Amino sugar and nucleotide sugar metabolism | 303 (1.42%) | ko00520 |
| 23 | Oxidative phosphorylation | 293 (1.37%) | ko00190 |
| 24 | ABC transporters | 283 (1.32%) | ko02010 |
| 25 | Phagosome | 256 (1.2%) | ko04145 |
| 26 | Nucleotide excision repair | 229 (1.07%) | ko03420 |
| 27 | RNA polymerase | 222 (1.04%) | ko03020 |
| 28 | Pyruvate metabolism | 217 (1.02%) | ko00620 |
| 29 | Phosphatidylinositol signaling system | 189 (0.88%) | ko04070 |
| 30 | Homologous recombination | 186 (0.87%) | ko03440 |
| 31 | Galactose metabolism | 185 (0.87%) | ko00052 |
| 32 | Stilbenoid, diarylheptanoid and gingerol biosynthesis | 184 (0.86%) | ko00945 |
| 33 | Cysteine and methionine metabolism | 183 (0.86%) | ko00270 |
| 34 | Circadian rhythm - plant | 182 (0.85%) | ko04712 |
| 35 | Cyanoamino acid metabolism | 177 (0.83%) | ko00460 |
| 36 | Phenylalanine metabolism | 168 (0.79%) | ko00360 |
| 37 | Fructose and mannose metabolism | 166 (0.78%) | ko00051 |
| 38 | Arginine and proline metabolism | 161 (0.75%) | ko00330 |
| 39 | Flavonoid biosynthesis | 158 (0.74%) | ko00941 |
| 40 | Limonene and pinene degradation | 158 (0.74%) | ko00903 |
| 41 | Peroxisome | 156 (0.73%) | ko04146 |
| 42 | DNA replication | 156 (0.73%) | ko03030 |
| 43 | Inositol phosphate metabolism | 155 (0.73%) | ko00562 |
| 44 | Zeatin biosynthesis | 150 (0.7%) | ko00908 |
| 45 | Other glycan degradation | 149 (0.7%) | ko00511 |
| 46 | Glutathione metabolism | 149 (0.7%) | ko00480 |
| 47 | Glycerolipid metabolism | 148 (0.69%) | ko00561 |
| 48 | Glyoxylate and dicarboxylate metabolism | 147 (0.69%) | ko00630 |
| 49 | Carbon fixation in photosynthetic organisms | 147 (0.69%) | ko00710 |
| 50 | Carotenoid biosynthesis | 145 (0.68%) | ko00906 |
| 51 | Ascorbate and aldarate metabolism | 141 (0.66%) | ko00053 |
| 52 | Aminoacyl-tRNA biosynthesis | 138 (0.65%) | ko00970 |
| 53 | Citrate cycle (TCA cycle) | 133 (0.62%) | ko00020 |
| 54 | Glycine, serine and threonine metabolism | 133 (0.62%) | ko00260 |
| 55 | Regulation of autophagy | 131 (0.61%) | ko04140 |
| 56 | Cutin, suberine and wax biosynthesis | 130 (0.61%) | ko00073 |
| 57 | alpha-Linolenic acid metabolism | 129 (0.6%) | ko00592 |
| 58 | Mismatch repair | 126 (0.59%) | ko03430 |
| 59 | Tyrosine metabolism | 125 (0.59%) | ko00350 |
| 60 | Valine, leucine and isoleucine degradation | 124 (0.58%) | ko00280 |
| 61 | Basal transcription factors | 123 (0.58%) | ko03022 |
| 62 | Base excision repair | 118 (0.55%) | ko03410 |
| 63 | Glycosylphosphatidylinositol(GPI)-anchor biosynthesis | 116 (0.54%) | ko00563 |
| 64 | Fatty acid metabolism | 115 (0.54%) | ko00071 |
| 65 | Pentose phosphate pathway | 113 (0.53%) | ko00030 |
| 66 | Terpenoid backbone biosynthesis | 113 (0.53%) | ko00900 |
| 67 | Protein export | 111 (0.52%) | ko03060 |
| 68 | Proteasome | 110 (0.51%) | ko03050 |
| 69 | Propanoate metabolism | 106 (0.5%) | ko00640 |
| 70 | Tryptophan metabolism | 102 (0.48%) | ko00380 |
| 71 | Porphyrin and chlorophyll metabolism | 100 (0.47%) | ko00860 |
| 72 | Sphingolipid metabolism | 100 (0.47%) | ko00600 |
| 73 | Photosynthesis | 98 (0.46%) | ko00195 |
| 74 | N-Glycan biosynthesis | 97 (0.45%) | ko00510 |
| 75 | Alanine, aspartate and glutamate metabolism | 96 (0.45%) | ko00250 |
| 76 | Nitrogen metabolism | 93 (0.44%) | ko00910 |
| 77 | Ubiquinone and other terpenoid-quinone biosynthesis | 91 (0.43%) | ko00130 |
| 78 | beta-Alanine metabolism | 89 (0.42%) | ko00410 |
| 79 | SNARE interactions in vesicular transport | 88 (0.41%) | ko04130 |
| 80 | Lysine degradation | 87 (0.41%) | ko00310 |
| 81 | Glycosaminoglycan degradation | 86 (0.4%) | ko00531 |
| 82 | Phenylalanine, tyrosine and tryptophan biosynthesis | 85 (0.4%) | ko00400 |
| 83 | Flavone and flavonol biosynthesis | 84 (0.39%) | ko00944 |
| 84 | Fatty acid biosynthesis | 83 (0.39%) | ko00061 |
| 85 | Steroid biosynthesis | 77 (0.36%) | ko00100 |
| 86 | Biosynthesis of unsaturated fatty acids | 73 (0.34%) | ko01040 |
| 87 | Fatty acid elongation | 69 (0.32%) | ko00062 |
| 88 | Natural killer cell mediated cytotoxicity | 68 (0.32%) | ko04650 |
| 89 | Isoquinoline alkaloid biosynthesis | 61 (0.29%) | ko00950 |
| 90 | Diterpenoid biosynthesis | 61 (0.29%) | ko00904 |
| 91 | Glycosphingolipid biosynthesis - ganglio series | 59 (0.28%) | ko00604 |
| 92 | Tropane, piperidine and pyridine alkaloid biosynthesis | 58 (0.27%) | ko00960 |
| 93 | Selenocompound metabolism | 55 (0.26%) | ko00450 |
| 94 | Valine, leucine and isoleucine biosynthesis | 55 (0.26%) | ko00290 |
| 95 | Circadian rhythm - mammal | 53 (0.25%) | ko04710 |
| 96 | Brassinosteroid biosynthesis | 52 (0.24%) | ko00905 |
| 97 | Linoleic acid metabolism | 51 (0.24%) | ko00591 |
| 98 | Pantothenate and CoA biosynthesis | 50 (0.23%) | ko00770 |
| 99 | Sulfur metabolism | 47 (0.22%) | ko00920 |
| 100 | Folate biosynthesis | 46 (0.22%) | ko00790 |
| 101 | Butanoate metabolism | 46 (0.22%) | ko00650 |
| 102 | Histidine metabolism | 45 (0.21%) | ko00340 |
| 103 | One carbon pool by folate | 45 (0.21%) | ko00670 |
| 104 | Riboflavin metabolism | 44 (0.21%) | ko00740 |
| 105 | Benzoxazinoid biosynthesis | 41 (0.19%) | ko00402 |
| 106 | Non-homologous end-joining | 40 (0.19%) | ko03450 |
| 107 | Arachidonic acid metabolism | 37 (0.17%) | ko00590 |
| 108 | Nicotinate and nicotinamide metabolism | 36 (0.17%) | ko00760 |
| 109 | Lysine biosynthesis | 33 (0.15%) | ko00300 |
| 110 | Isoflavonoid biosynthesis | 30 (0.14%) | ko00943 |
| 111 | Photosynthesis - antenna proteins | 29 (0.14%) | ko00196 |
| 112 | Monoterpenoid biosynthesis | 28 (0.13%) | ko00902 |
| 113 | Other types of O-glycan biosynthesis | 26 (0.12%) | ko00514 |
| 114 | Glycosphingolipid biosynthesis - globo series | 26 (0.12%) | ko00603 |
| 115 | Glucosinolate biosynthesis | 24 (0.11%) | ko00966 |
| 116 | Vitamin B6 metabolism | 23 (0.11%) | ko00750 |
| 117 | Taurine and hypotaurine metabolism | 22 (0.1%) | ko00430 |
| 118 | Sulfur relay system | 20 (0.09%) | ko04122 |
| 119 | Thiamine metabolism | 19 (0.09%) | ko00730 |
| 120 | Sesquiterpenoid and triterpenoid biosynthesis | 17 (0.08%) | ko00909 |
| 121 | Indole alkaloid biosynthesis | 15 (0.07%) | ko00901 |
| 122 | Synthesis and degradation of ketone bodies | 14 (0.07%) | ko00072 |
| 123 | Biotin metabolism | 10 (0.05%) | ko00780 |
| 124 | C5-Branched dibasic acid metabolism | 10 (0.05%) | ko00660 |
| 125 | Anthocyanin biosynthesis | 8 (0.04%) | ko00942 |
| 126 | Lipoic acid metabolism | 7 (0.03%) | ko00785 |
| 127 | Caffeine metabolism | 5 (0.02%) | ko00232 |
| 128 | Betalain biosynthesis | 2 (0.01%) | ko00965 |
